# Catalytically active guanylyl cyclase-B requires glycosylation and mutations that inhibit this process cause dwarfism

**DOI:** 10.1186/2050-6511-16-S1-A44

**Published:** 2015-09-02

**Authors:** Deborah M Dickey, Aaron B Edmond, Thomas Chaffee, Lincoln R Potter

**Affiliations:** 1 Department of Biochemistry, Molecular Biology and Biophysics, University of Minnesota, Minneapolis, MN, USA

## Background

C-type natriuretic peptide (CNP) is a paracrine factor that stimulates long bone growth, axonal path finding and inhibits meiosis in the oocyte [1]. The biologic signaling receptor for CNP is guanylyl cyclase (GC)-B, also known at NPRB or NPR2 [2].

GC-B is a homo-oligomer, possibly a dimer, containing a glycosylated extracellular ligand-binding domain, a single membrane-spanning region, and intracellular kinase homology domain (KHD), dimerization domain and C-terminal GC catalytic domain. Phosphorylation of the region leading into and at the beginning of the kinase homology domain is required for CNP activation of GC-B and dephosphorylation inactivates the enzyme [3].

Homozygous inactivating mutations in GC-B result in Acromesomelic Dysplasia, Type Maroteaux (AMDM) dwarfism [4-6], and heterozygous inactivating mutations in GC-B cause non-pathological reductions in stature [7]. Conversely, genetic mutations that increase GC-B activity result in skeletal overgrowth [8-10].

More than fifteen inactivating missense mutations in GC-B have been identified in humans. These mutations are randomly distributed from the N-terminus (P32) to the C-terminus (G959A) of the enzyme, consistent with two potential mechanisms. The first involves multiple processes like disruption of CNP or GTP binding to the extracellular or catalytic domains, respectively. The second more general mechanism involves conformational changes in secondary, tertiary or quaternary structure that preclude catalytic domain formation or activation.

Previous investigators reported that 11 out of 12 [1], 2 out of 3 [2] or 1 out of 2 [3] missense mutations inhibited the transport of GC-B to the cell surface due to defective cellular trafficking and retention in the ER as indicated by reduced immunofluorescence imaging. Thus, the current hypothesis is that AMDM mutations inactivate GC-B by disrupting intracellular trafficking. Here, we report that four intracellular GC-B mutants known to cause AMDM dwarfism bind CNP on the cell surface but have dramatically reduced catalytic activity.

## Results

**Figure 1:**
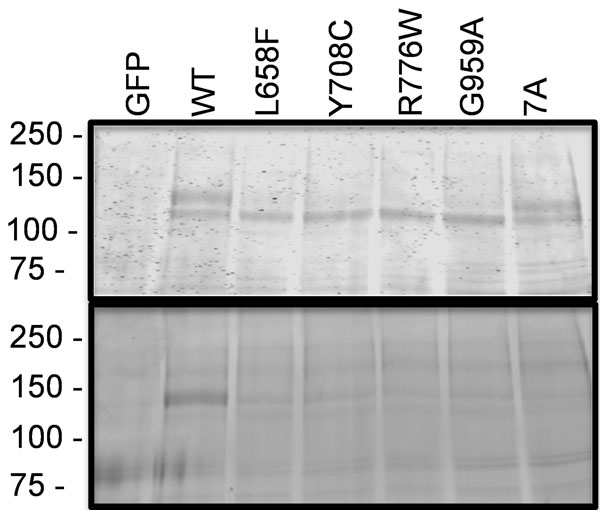
**AMDM mutants are not processed to the fully glycosylated and phosphorylated form of GC-B.** WT and the indicated missense versions of GC-B were isolated from transiently transfected 293T cells by sequential immunoprecipitatin/SDS-PAGE purification. The resulting gel was incubated with ProQ Diamond dye to determine GC-B phosphate levels (lower panel). The same gel was then washing and stained with Coomassie to determine the amount of GC-B protein present (upper panel). MW in Kilo-Daltons is shown for protein standards on left. Abbreviations are: GFP, green fluroscent protein; 7A, GC-B containing alanine substitutions for all seven known phosphorylation sites.

*AMDM* mutants are incompletely processed- Post-or co-translational processing of the four intracellular AMDM causing mutants (L658F, Y708C, R776W and G959A) was compared against WT-GC-B and GC-B-7A, a mutant containing alanine substitutions for all known phosphorylation sites [3], in reduced SDS gels containing immunoprecipitated receptors isolated from transiently transfected 293T cells. Coomassie staining (Fig. 1, upper panel) of WT-GC-B revealed a slower migrating, diffuse species (top band) that is maximally glycosylated and phosphorylated and a faster migrating species (bottom band) that is incompletely glycosylated and is not phosphorylated [3-14]. The upper band was also present in samples from GC-B-7A indicating that changes in phosphorylation does not account for the difference in migration of the two bands. In contrast, all four AMDM mutants had severe reductions in the slower migrating, fully glycosylated and phosphorylated species, but had normal amounts of the faster migrating, incompletely glycosylated species. ProQ Diamond staining, a dye that binds GC-B in phosphate-dependent manner [5], of the same gel indicated that only the slower migrating species of the WT receptor was phosphorylated (Fig. 1, lower panel).

**Figure 2:**
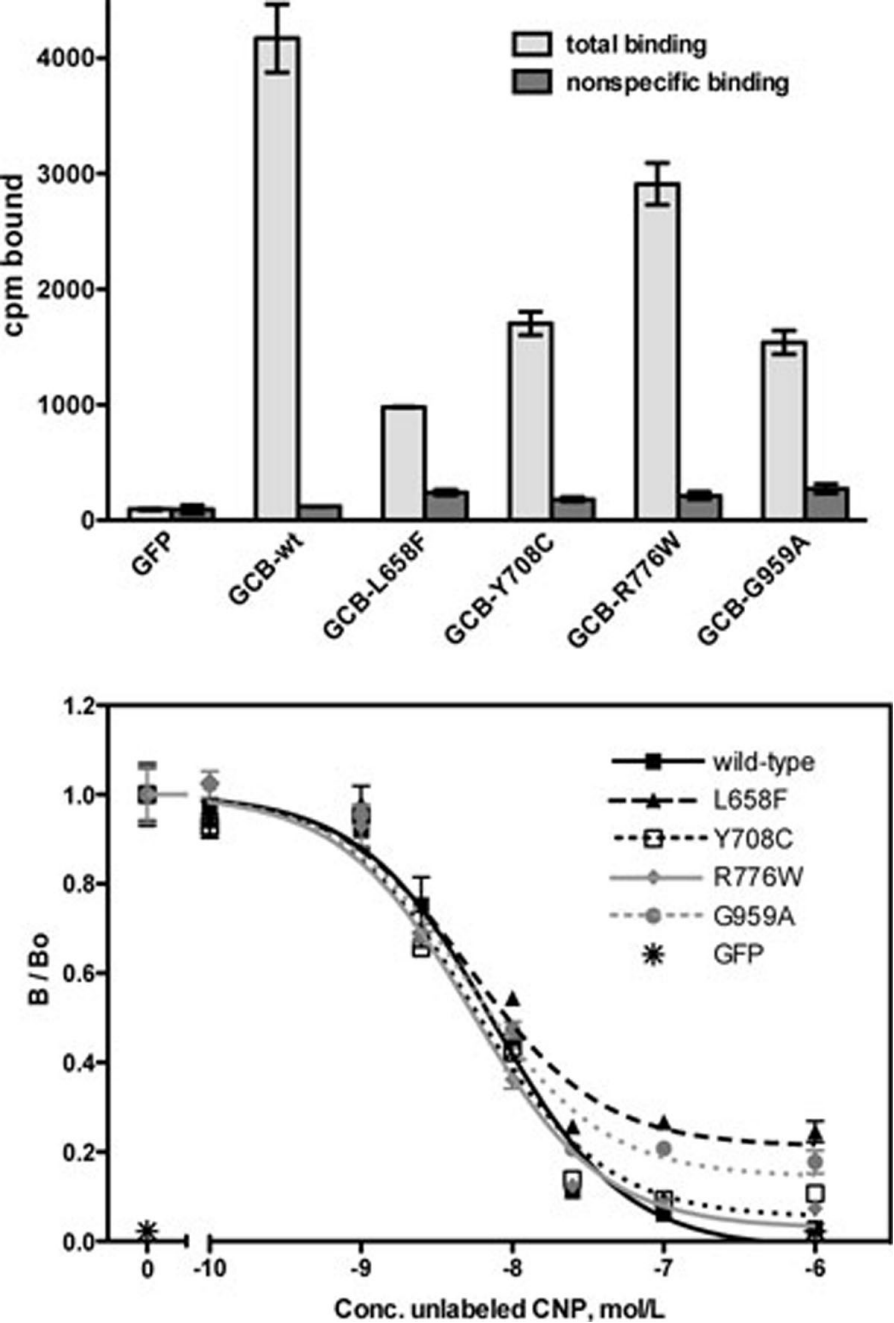
**The AMDM mutants are on the cell surface and bind CNP like WT-GC-B.** 293 cells were transiently transfected with plasmids containing green fluorescent protein (GFP), WT-GC-B or the indicated mutants. Cells were incubated for 1 hr at 4°C with 50 pM ^125^I-CNP in the presence or absence of 1 μM (upper panel) or increasing concentrations (lower panel) of non-radioactive CNP. The cells were rinsed with binding medium to remove non-specific ^125^I-CNP, removed from the plate with NaOH, and subjected to gamma counting to determine the amount of specifically bound 125I-CNP associated with the cells. The vertical arrows in the bars and symbols represent the SEM where n = 3.

The AMDM GC-B Mutants Are On the Cell Surface and Bind CNP- 125I-CNP binding assays were performed on live 293T cells transfected with the individual AMDM mutants, WT-GC-B as a positive control, and GFP as a negative control (Fig. 2, upper panel). Low radiation levels in GFP transfected cells and in GC-B expressing cells incubated with excess non-radioactive CNP (nonspecific binding), confirmed that the binding was specific for GC-B. All four mutants specifically bound 125I-CNP, consistent with GC-B being on the cell surface. Total 125I-CNP binding to each mutant was variable and less than that observed for the WT receptor, which is expected since the mutants lack the upper completely processed species only seen in WT-GC-B (Fig. 1, upper panel). Competition binding assays indicated that the affinity of each mutant for CNP was similar to that of the WT-GC-B (Fig. 2, lower panel). These data indicate that all four mutants are on the cell surface and that glycosylation of GC-B is neither required to bind CNP nor required for GC-B to adopt a conformation capable of binding CNP.

**Figure 3:**
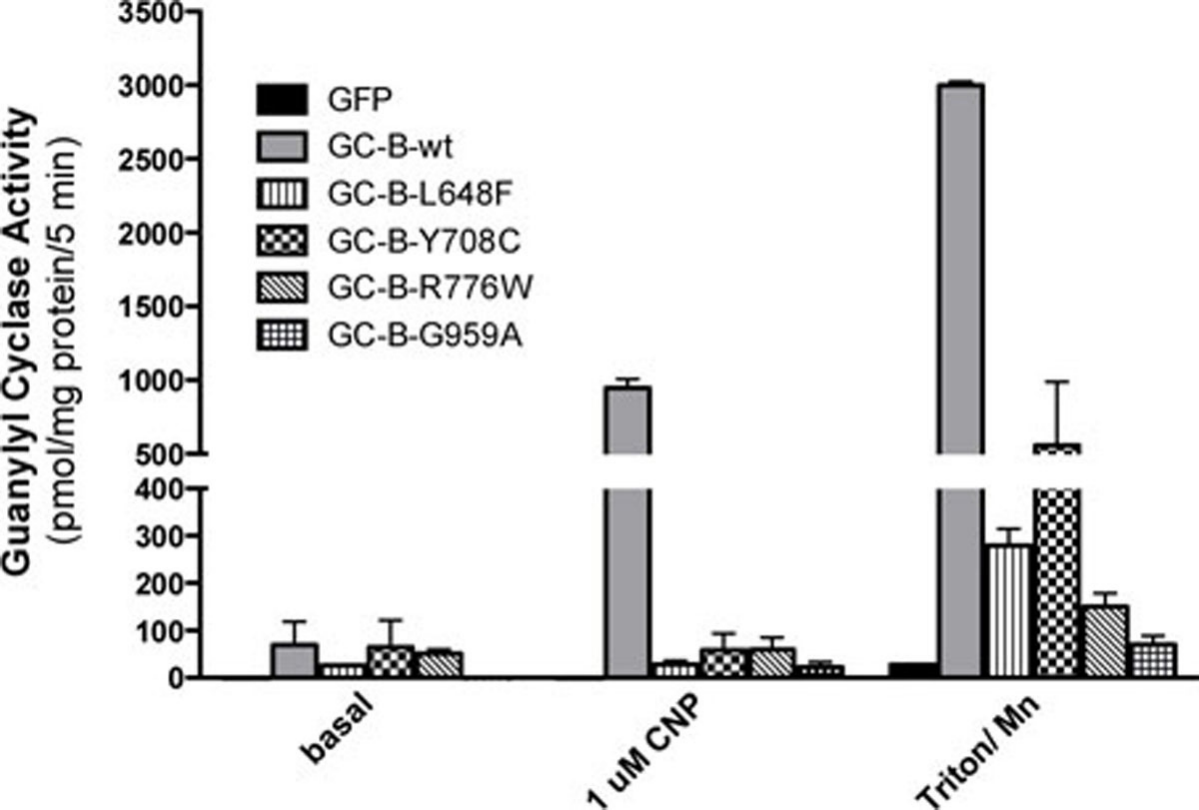
**The AMDM mutants have markedly reduced guanylyl cyclase activity.** 293 cells were transiently transfected with plasmids containing green fluorescent protein (GFP), WT-GC-B or the indicated mutants of GC-B. Crude membranes were prepared from the transfected cells and assayed for guanylyl cyclase activity under basal, CNP-stimulated or Triton X-100 and manganese-stimulated conditions. The vertical arrows in the bars and symbols represent the SEM where n = 3.

AMDM mutants have dramatically reduced GC activity-The ability of the GC-B mutants to form active catalytic domains that can be stimulated by CNP or non-ionic detergent and manganese was tested in membranes from transfected 293 cells (Fig. 3). GC activity was measured under basal (Mg2+GTP and ATP), CNP-stimulated (Mg2+GTP, ATP, CNP) and artificial (Mn2+GTP and Triton X-100) conditions. The latter activates the enzyme independently of natriuretic peptide or phosphorylation and is an excellent indicator of total catalytic domain formation [3]. No significant differences were observed under basal condition but GC activities of the mutant enzymes measured under CNP- and detergent conditions was less than 20% of the activity observed for WT-GC-B. Since the AMDM mutations inhibit activity in the presence of detergent and CNP, it suggests that catalytic domain formation is inhibited as opposed to inhibiting CNP-dependent activation of a preformed catalytic domain.

## Conclusion

We conclude that glycosylation of GC-B is required for active catalytic domain formation and that the majority of GC-B mutations that reduce stature inactivate the enzyme by decreasing receptor glycosylation not by inhibiting trafficking to the plasma membrane.
